# Attention Deficit Hyperactivity Disorder Assessment Based on Patient Behavior Exhibited in a Car Video Game: A Pilot Study

**DOI:** 10.3390/brainsci12070877

**Published:** 2022-07-01

**Authors:** Aaron Sujar, Sofia Bayona, David Delgado-Gómez, Carolina Miguélez-Fernández, Juan Ardoy-Cuadros, Inmaculada Peñuelas-Calvo, Enrique Baca-García, Hilario Blasco-Fontecilla

**Affiliations:** 1 Department of Computer Engineering, Universidad Rey Juan Carlos, 28933 Madrid, Spain; aaron.sujar@urjc.es (A.S.); sofia.bayona@urjc.es (S.B.); 2 Department of Psychiatry, Puerta de Hierro University Hospital, Health Research Institute Puerta de Hierro-Segovia de Arana (IDIPHISA), 28222 Majadahonda, Spain; hmblasco@yahoo.es; 3 Center for Computational Simulation, Universidad Politécnica de Madrid, 28040 Boadilla del Monte, Spain; 4 Department of Statistics, Universidad Carlos III, 28903 Getafe, Spain; 5 Department of Child and Adolescent Psychiatry, Hospital Niño Jesús, 28009 Madrid, Spain; carolina.miguelezfer@gmail.com; 6 Department of Psychology, Faculty of Health Sciences, University Rey Juan Carlos, Avda. Atenas s/n, 28922 Alcorcón, Spain; juan.ardoy@urjc.es; 7 Department of Child and Adolescent Psychiatry, University Hospital 12 de Octubre, 28041 Madrid, Spain; inmaculada.penuelas@salud.madrid.org; 8 Department of Psychiatry, IIS-Jimenez Diaz Foundation, 28040 Madrid, Spain; ebacgar2@yahoo.es; 9 CIBERSAM (Centro de Investigación en Salud Mental), Carlos III Institute of Health, 28029 Madrid, Spain; 10 Department of Psychiatry, Universidad Autónoma de Madrid, 28049 Madrid, Spain; 11 ITA Mental Health, 28043 Madrid, Spain

**Keywords:** ADHD, video games, e-health, inattention, hyperactivity, attention deficits, attention span, behavioral patterns, neurodevelopmental disorders

## Abstract

Symptoms of Attention Deficit Hyperactivity Disorder (ADHD) include excessive activity, difficulty sustaining attention, and inability to act in a reflective manner. Early diagnosis and treatment of ADHD is key but may be influenced by the observation and communication skills of caregivers, and the experience of the medical professional. Attempts to obtain additional measures to support the medical diagnosis, such as reaction time when performing a task, can be found in the literature. We propose an information recording system that allows to study in detail the behavior shown by children already diagnosed with ADHD during a car driving video game. We continuously record the participants’ activity throughout the task and calculate the error committed. Studying the trajectory graphs, some children showed uniform patterns, others lost attention from one point onwards, and others alternated attention/inattention intervals. Results show a dependence between the age of the children and their performance. Moreover, by analyzing the positions by age over time using clustering, we show that it is possible to classify children according to their performance. Future studies will examine whether this detailed information about each child’s performance pattern can be used to fine-tune treatment.

## 1. Introduction

Attention Deficit Hyperactivity Disorder (ADHD) is one of the most common neurodevelopmental disorders in childhood and adolescence, with an estimated prevalence of 7.2% [1]. This mental condition is characterized by having excessive activity, difficulties in maintaining attention, and inability to act reflectively [2,3,4].

Some researchers understand ADHD as a developmentally impaired executive functions [3]. For example, working memory (short-term memory and central executive in verbal and visuospatial domains) has also been related to the attention symptoms [5]. Willcutt et al. argue that moderate effect sizes and lack of universality of executive functions deficits in ADHD indicate that executive functions weaknesses are neither necessary nor sufficient to cause all cases of ADHD [6]. Other studies suggest that there is a relationship between brain structure and neurodevelopmental disorders. For example, Shaw et al. [7] suggest that ADHD is characterized by a delay in cortical maturation. Castellanos et al. [8] found that people with ADHD have less brain volume (approximately 3.2%). Also, the role of dysfunctional prefrontal cortical activity in neuropsychiatric disorders, and how this influences cognitive and emotional processes in different psychopathologies have been studied [9,10]. Battaglia et al. [11] even suggest that defects in the mentioned brain areas may affect emotional learning, such as fear responses.

Although neurodevelopmental disorders (NDDs) are widely presumed to be inherited [12], other studies suggest that several environmental risk factors may be involved [13]. For example, Carlsson et al. [14] enumerate that low birth weight, gestational age, and low family income or transient income decline during childhood are associated with ADHD. Additionally, ADHD presents a lot of comorbidity with other disorders [15,16]. For example, anxiety disorders increase the severity of the condition [17], and ADHD was found to be present in 30–40% of epileptic children [18].

The symptoms of ADHD negatively impact all areas of the person’s life. Higher rates of school failure, a greater probability of suffering addiction problems, or a higher accident rate are found among the ADHD consequences [19,20]. Impulsivity-related disorders are characterized by poor regulation and control that may worsen in the presence of emotional signals [11]. Besides, social and emotional impairment are present too in children and adolescents with ADHD [21]. Moreover, ADHD not only impacts the quality of life of the patients who suffer from it, but also that of their families [22]. On top of that, the COVID-19 pandemic has worsened the consequences caused by ADHD. For example, some studies have shown a worsening of the symptom severity in children with ADHD, and an increase in the stress level of their parents [23,24].

Fortunately, the severity and persistence of the above symptoms can be significantly reduced if the disorder is identified early and appropriate and individualized treatment is applied. However, obtaining an early and accurate diagnosis of ADHD remains a major problem in clinical psychology and psychiatry. Although efforts are being made to develop precision diagnostics (searching for biomarkers that may be related to those impairments that affect cognitive functions [13,25]), currently, the diagnosis of ADHD is made through a clinical interview in which the medical professional relies on scales filled out by caregivers and teachers. This means that the diagnosis of ADHD depends on the training, experience and knowledge of the medical professional, as well as on the observation skills of the caregivers. Several authors have criticized the manner in which ADHD is diagnosed given its subjective nature [26,27]. This fact can be seen in the results of a study in which 473 psychotherapists, specialized in children and adolescents, committed more than 20% of false negatives and 15% of false positives [28].

With the aim of improving the accuracy in the diagnosis of ADHD, several expert systems are being developed [29,30,31]. These systems aim to obtain an accurate characterization of ADHD through the identification of new predictors and the application of techniques recently developed in data science [32,33]. Within the different research lines that are being carried out, one that is receiving considerable attention is the analysis of the behavior exhibited by the patient while performing computerized tests and video games. These tests obtain several predictors such as the reaction time or the number of omissions and commissions, which have been related to inattention and impulsivity [34,35]. A shortcoming of these tests, however, is that they do not analyze the patient’s behavior during the entire test but only at certain instants where the patient has to perform or inhibit certain actions.

Most of the published papers attempt to classify participants into children with or without ADHD. Our work, however, focuses on providing more information on cases that have already been diagnosed, for a deeper understanding of the child’s behavior that could enable further tailoring and personalization of treatment. In this article, a system is developed to objectively observe how the symptoms of ADHD patients affect their performance in a video game task. The system uses video game technology to record objective measurements while children perform a driving task in a video game. Unlike other works, we register the participant’s activity continuously, obtaining considerably more information for its subsequent statistical analysis. Our aim is to study the children’s performance along the test and the different performance patterns that appear. We hypothesize that it is possible to study when children lose attention, how long they are able to maintain focus on the task, and to classify children according to their different behaviors.

The structure of this article is as follows. Section 2 presents the published works most relevant to this research and highlights the differences and value of our proposal. After that, the methods and the developed system are described in Section 3. Section 4 shows the results obtained in the experiment aimed at evaluating the proposed system. The article concludes with Section 5 and Section 6, where we discuss the findings and limitations, propose future lines of research, and conclude.

## 2. Related Work

The interest in providing objective tests to aid in the diagnosis of ADHD is not new. Continuous performance test solutions are based on using technology to be able to collect predictors objectively without relying on human judgment. Among the computerized tests, one of the most widely used is the Conners’ Continuous Performance Test (CPT) [34], in which the examinee must press the space bar on a keyboard as fast as possible each time certain characters appear on a computer screen. If a forbidden character, referred to as an X-stimulus, appears on the screen, examinees must inhibit their reaction. This method is known as the go/no-go paradigm [36], where the participant has to show a specific reaction each time certain stimuli occur and inhibit any reaction when other less frequent stimuli appear. Several studies have demonstrated that these predictors have the ability to differentiate children with and without ADHD [37].

Based on this task, video game-based continuous performance tests have been developed. One advantage of using video games is that children find them more fun, palliating a possible aversion to classic tests and facilitating a more natural approach. Another is that the game distracts from the assessment objective, making it more difficult for children to engage in intentional behaviors such as faking or trying to guess an appropriate answer on a questionnaire. A first approach is to use commercial video games to perform diagnostics by analyzing user behavior [38,39]. The main problem with using proprietary video games is that medical professionals cannot choose the variables to collect or their degree of accuracy. A common approach is to adapt current tests to children by replacing the stimulus with cartoons to prevent reading problems [40] or idiomatic problems [35]. Regarding other tests and scales, Crepaldi et al. developed a game that includes variations of the go/no-go and Stroop tests recording impulsivity, omissions, anticipation, and position errors [41]. In a different work, Delgado-Gómez et al. study found a significant correlation between the results of the SWAN scale and the measures registered during an infinite runner-based video game [42]. Furthermore, other works investigated the possibility of using games to assess other aspects of ADHD such as stress [43] or prosocial behavior [44]. For more examples, please refer to this recent systematic review [33].

Regardless of the format of the test and the technology used, most of these tests are oriented to support diagnosis by registering the number of hits, omissions, commissions, and reaction times as predictors of inattention and hyperactivity. Some solutions incorporate tracking devices [45,46,47,48], measure pupil size [49] or attention-related eye vergence [50], or even register electroencephalogram (EEG) to detect ADHD [51]. Other works use virtual reality so that children can perform a CPT on the blackboard of a virtual classroom [52,53,54,55].

The present work, despite the use of a video game, is not focused on the ADHD diagnosis itself, but proposes a system that records information that enables to study in detail the behavior displayed by children already diagnosed with ADHD during a car-driving video game. Our solution can continuously record the car trajectory along time to look for patterns. This system has been specifically designed so that the game can be deployed online to be accessible to everyone without requiring any specific hardware.

## 3. Material and Methods

This section presents the system which collects data continuously while subjects perform a driving task in a video game, as well as all the details of the experiment. The system is widely affordable, as the only hardware requirement is a computer.

### 3.1. Set-Up

**Figure 1:**
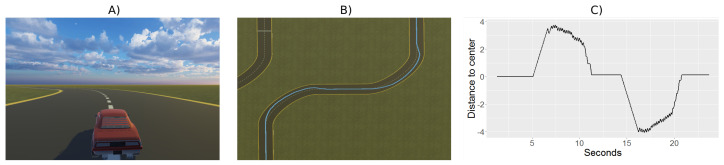
Car driving video game. (**A**) User view. (**B**) Example of a recorded trajectory (in blue). (**C**) Error graph over time.

The video game for the test is based on classic driving games, where the user drives a car on a road with curves and straights. The car moves with a constant speed along the route and it receives a force in the curves that moves it away from the center line, forcing users to quickly correct their position. The user’s goal is to keep the car as close to the center line of the road as possible, see panel (A) in Figure 1. The test duration was set to 11.5 min, because the ability to maintain sustained attention in a person without any condition ranges from 10 to 20 min [56]. Therefore, it is to be expected that patients with ADHD will have a lower attention span.

During the test, the child should ideally keep the car on the center line of the road (Figure 1A). The position of the car is recorded as shown in Figure 1B, where the continuous blue line represents the followed trajectory. The error, measured as the distance from the trajectory to the center of the road at each time instant, will be plotted and analyzed to characterize the behavior of the participants (see Figure 1C). In this example, we can see that at the beginning, from seconds 0 to 4, the error committed has been close to zero (the car was following the center of the road very well). Then, the car deviated upwards (from seconds 4 to 10) and returned to the center line. It then drifted downwards (from seconds 13 to almost 20), at which point it managed to return again to the center line, making very little error from second 20 onwards.

The game has been created using the Unity 3D tool [57]. A circuit was built using a road construction plugin called EasyRoad3D [58]. The circuit, designed to be completed in 11.5 min, contains a total of 58 curves and 58 straights. All curved and straight road sections are equal. All curves are 90 degrees. The car could not leave the track and remained on the edge of the road if there was no user intervention.

The user could move the car laterally with a force of 10 m per second with the arrow keys. However, when taking curves, the car experienced a force towards the outside of the curve (equivalent to 0.45 of the lateral displacement force) which meant that the user had to pay attention to correct the deviation.

In order to have the same number of samples for all users, we took 50 samples per second up to a total of 34,496 samples per participant. The following parameters are collected: position of the car on the road, direction of the road (straight, left curve, right curve), button pressed (left key, right key or none) and whether there was collision with the outside of the curve.

Before performing the actual task, participants practiced with a 40-s tutorial, in which the route consisted of 4 curves and 4 straights equal to those of the test, so that the user could get used to it during that time. The data collected in the tutorial was not used for the results.

Next, we will detail the sample of our experiment, the ethical procedure, the experiment set-up, and the statistical analysis.

### 3.2. Sample

The sample in our experiment consisted of a group of 63 children (69.84% of males) diagnosed with ADHD according to DSM-5 criteria attending the Psychiatry Department of Hospital Puerta de Hierro (Majadahonda, Spain). The mean and standard deviation of the age was 12.89 and 2.80, respectively. The minimum age was 7, and the maximum was 17 (See Table A1).

The consent form and the study protocol were reviewed and approved by the Institutional Review Board of Hospital Puerta de Hierro of Madrid (H.U.P.H.: PI 178/19). Once the test was explained in detail, caregivers were asked to sign the informed consent form. Participants were assigned an identification number to maintain their anonymity and were conducted to a room where a computer was running the game.

### 3.3. Protocol

First, a researcher explained the instructions of the game so that the child would understand the game’s mechanics and could test how the car works practicing with a 40-second tutorial. Subsequently, the child performed the test by driving the car through 58 curves and 58 straights. After finishing, participants were asked to fill out a brief satisfaction questionnaire, giving their opinion on the experience. A total of 57 children filled this questionnaire.

### 3.4. Satisfaction Questionnaire

The questionnaire is composed of the four questions shown in Table 1. The first question asked to rate the game from one to ten. Then, children have to fill in two free-text questions about what they liked most and least about the test. In addition, there was a fourth question asking explicitly whether they found the test long.

### 3.5. Statistical Analysis

A descriptive analysis of the results, studying the effect of the age of the participants on the error via multiple box plots was performed.

We compare error graphs for different participants, to observe the evolution of the error over time and apply the *k*-means unsupervised machine learning algorithm to classify the participants according to their errors. For this purpose, we use a scree plot to determine the most reasonable number of clusters in each case.

Once the groups were made and those children with different behavior were identified, we used outlier identification to determine the moments when attention was lost and analyze the different patterns.

We want to disregard occasional atypical positions lasting only an instant, so we consider that an individual starts to lose attention when errors occur that last a second or more. This duration of one second has been established empirically for this work and will have to be determined for each task. Observation of the trajectory graphs with sustained errors can serve to determine the moments when participants lose and regain (if at all) attention.

In the following section, we present the results obtained from the different analyses performed, detailing each of them.

## 4. Results

**Figure 2:**
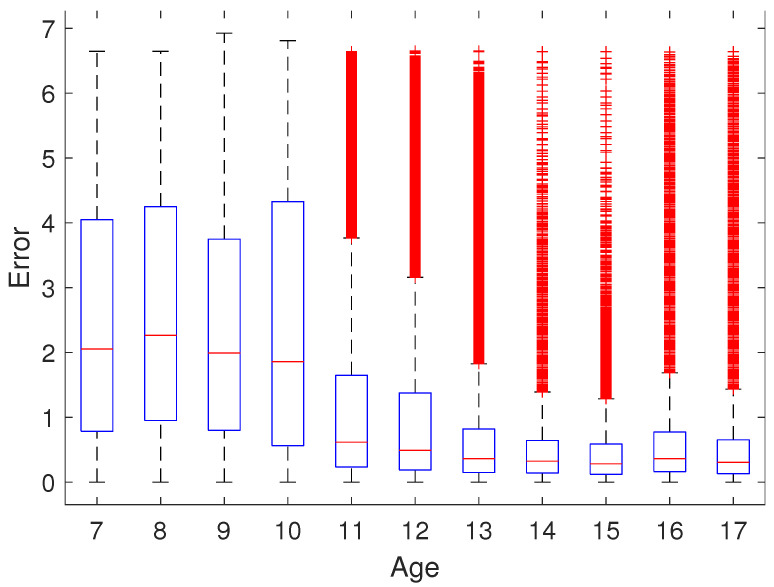
This graph shows the absolute error of the 34496 car positions registered for each participant, by age.

First, we analyze the influence of the age of the participant on the error committed with a multiple box plot. As a measure of error, the distance from the car to the centerline of the road was used. For each participant, this measurement was captured at a frequency of approximately 1/50. The results obtained for the absolute error are shown in Figure 2, where we can observe that the error median is higher for younger children and is quite similar after 13 years of age. Please, note that although, in some cases, there are quite a number of outliers (marked in red), all of them could belong to one or a few participants, since all the 34,496 positions of each participant are taken into account. It can also be observed that the error variability decreases with age.

**Figure 3:**
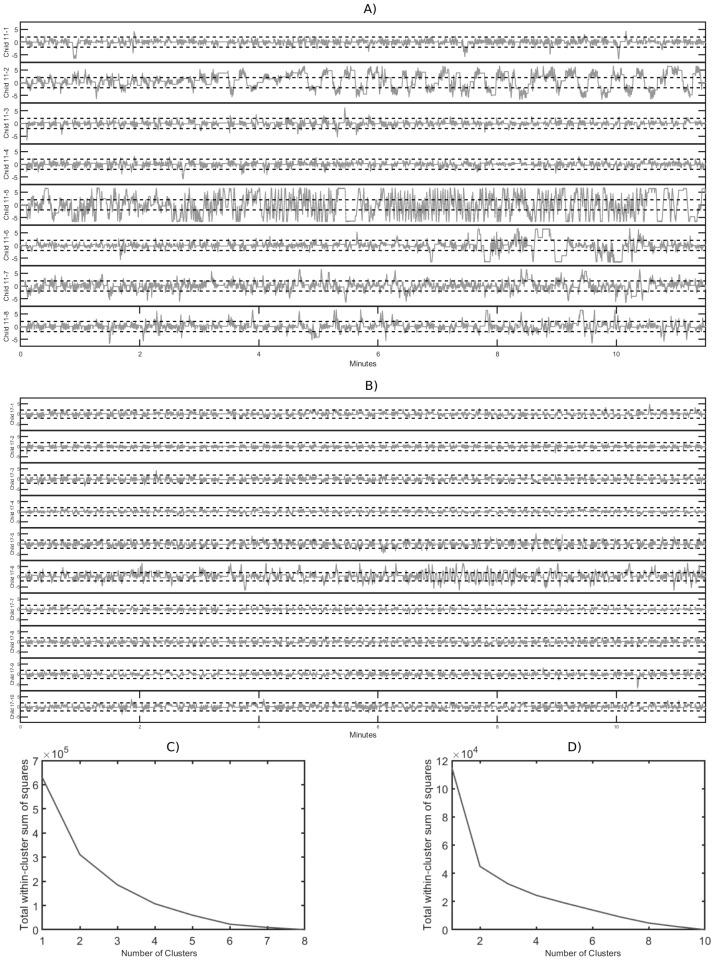
A *k*-means cluster analysis was performed. (**A**) The error graph over time for 11-year-olds. (**B**) The error graph over time for 17-year-olds. (**C**,**D**) show the scree plots with the number of clusters for 11-year-olds and 17-year-olds respectively.

Observing the error graphs comparing different individuals, we can extract considerable information. Figure 3A shows the error graphs of the 11-year-olds, while Figure 3B corresponds to those of the 17-year-olds. As in Figure 2, we can observe that both the error and its variability are higher for younger subjects.

One can also see at a glance which subjects made a major error. In the graphs of the 11-year-olds, the child labeled 11-5 makes the biggest error. In the case of the 17-year-olds, the child labeled 17-6 clearly makes the most errors, both at the beginning and especially from almost the seventh min on.

It is very interesting to note that there are children who start the test quite well and, from a certain point (in the case of the child labeled 11-2, around the third min and a half) until the end, their performance is much worse. Another pattern is show by the child labeled 11-6, who starts well, then makes errors for time interval, and then seems to refocus, alternating intervals of little error with intervals of more error.

To classify children, a *k*-means cluster analysis was performed. Figure 3C,D show the scree (or elbow) plots, so that the most parsimonious balance between minimizing the number of clusters and minimizing the variance within each cluster can be chosen. The scree plots suggest three clusters for the 11-year-olds performance and two clusters for the 17-year-olds performance.The scree plots suggest three clusters for the performance of 11-year-olds and two clusters for the performance of 17-year-olds.

Looking in detail which participants belong to each group, for the 17-year-olds, the *k*-means algorithm separates the child labeled 17-6 into one cluster and all other children into the other group.

The classification of the 11-year-olds, when grouping them into three clusters is as follows. One cluster consists of the child labeled 11-5, who makes quite a few errors throughout the entire test. Another cluster includes the child labeled 11-2, who performed well up to a certain point (around the third min and a half) and thereafter seems to become distracted or unable to sustain attention, leading to a much worse performance and more errors until the end. The third group comprises the remaining children who make fewer errors.

If we had classified the children into four groups instead of three, the child labeled 11-6 would also have been in a separate group. The child performs correctly at the beginning of the test, then makes quite a few errors over time (centered on minutes 8 to 11), and then seems to regain concentration to make fewer errors at the end (from minute 11 to the end).

**Figure 4:**
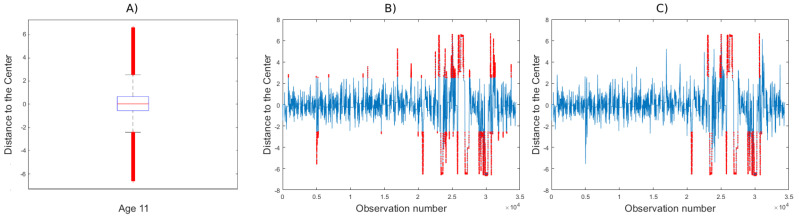
Example of attention span analysis of participant 11-6. (**A**) Boxplot of distance to the center of 11-year-olds to determine outliers. (**B**) Graph showing in red outlier positions of the car in participant 11-6’s recorded trajectory. (**C**) In this graph, only errors that were sustained for more than one second are colored in red.

Once the children have been classified into the different groups, it is easy to identify those with a different behavior. In order to observe their attention pattern, we propose a procedure to determine when each participant loses/recovers attention with respect to his or her group of age. As an operational example, we will show the case of participant 11-6. Figure 4A shows the boxplot with the car positions for all children of that age, showing the outliers in red. These outliers are those observations with a value under Q1 − 1.5 IQR or with a value higher than Q3 + 1.5 IQR, being Q1 and Q3 the first and third quartiles respectively, and IQR the interquartile range. For this age, a non-outlier position interval of [−2.4, 2.6] for 11-year-olds is established. Figure 4B shows the trajectory positions of participant 11-6, with outlying positions marked in red. However, to study attention during this video game task, we will discard instantaneous punctual errors and only consider those errors that are committed for more than one second (sustained errors). Figure 4C shows this type of sustained errors in red. We observe that for this child sustained errors start from observation 20,617, i.e., after 6.87 min. Attention was recovered from observation 31,008, i.e., after 3.43 min of inattention and then it was maintained until the end of the task.

Satisfaction questionnaire are shown in Table 1. The first columns shows how participants rate the videogame. Results show a positive evaluation of the video game with a score of 7.81 out of 10, (SD: 2.09). Regarding the questions about what did they like and what they did not like, they were free-text questions, so we grouped the answers into categories as they appeared according to their frequency as s shown below:

- To play a video game: children wrote that they liked or not the fact of playing a video game.
- Car Theme: this category groups answers about whether children liked that the theme of the game was about driving a car.
- Duration time: some of the users complained about the length of the test.
- Lack of realism: some of the children expressed that they would have preferred to have more control of the car (to be able to use the brake, to be allowed to drive freely or to deal with collisions).
- No answer.

As for the positive aspects, participants positively valued the fact that the test consisted of playing a video game (Video game 38.60%) and other 42.10% of the participants liked that it was about a car game (Car Theme). On the other hand, as a negative aspect, we find the duration as their major criticism (35.09%), although part of the sample (22.81%) does not like the car theme. Some children (3.51%) complain about they want to drive freely and that the car does not crash with the edge. Criticism of the time spent is also reflected in the next question, which shows that a majority of participants rated the test as long (59.65%).

## 5. Discussion

According to the literature, it is common for ADHD to present problems in maintaining attention, and therefore, when performing a task, this will impact the correctness and the reaction time. The theme of our video game, driving a car without distractions, was deliberately chosen with the intention of avoiding introducing emotional biases that could affect reaction times [11]. No rewarding gamification elements were introduced to minimize the risk for video game addiction [59].

Some studies show that there is a gender bias that has been observed when diagnosing false positives (there was more overdiagnosis when the gender was male [28]). Although this work does not focus on diagnosis, video game methods allow to obtain in-depth information on each subject avoiding this bias.

According to Biederman et al., maturity helps to improve ADHD symptoms and, therefore, performance is expected to be better the older the subject is [60]. The results of our study confirm this since, as shown in Figure 2, errors are strongly age-dependent.

As explained, the observation and analysis of children’s behavior on the test over time convey very interesting information. First, it allows us to look at error not only as a cumulative measure, but also to see at what times it is committed and how long it lasts. For example, the participant labeled 11-6 seems to have an attention span close to 7 min (see Figure 4C, while the attention span of the participant labeled 11-4 seems to be longer than 11.5 min, the test duration (see Figure 3A).

Furthermore, error graphs can be used to classify children into different groups. Some children keep a uniform performance over time (either with a high error or a low error). Other children, however, make little error until a given point when they lose concentration, and yet another pattern occurs where children start with little error and then alternate time intervals with many errors and others with few errors. This last pattern suggests that the participant maintains attention for a time time interval, then loses it, refocuses, gets distracted again, and so on.

The proposed detailed study of childrens’ behavior would, if sufficient data are collected, enable a characterization of the average attention span for an age group. In addition, for each child, we could study whether the child tends to have similar patterns in different performances or whether a child’s pattern in one test differs from his or her pattern in other tests.

Regarding the satisfaction questionnaire results, children rated the test with 7.81 out of 10. There were more than a third of the children who liked that it was a video game. Also, some of the participants (42.10%) liked the theme of driving a car (compared to 22.81% who did not like it). For future studies, we could consider adapting the theme to the tastes and preferences of the children. In open-ended question 3, 35.09% of the participants said that they did not like the length of the test, and 59.65% of the children said that they found it long. This result is to be expected since we chose a test duration of 11.5 min precisely so that it would be hard for the participants to maintain their attention. For subsequent tests with the same participants, once their particular attention span is known, we could consider adapting the duration of the test so that it would be enough for the research purpose but not too long.

### Limitations and Future Directions

A limitation of the study is the sample size. A consequence of this is that results include different age ranges, which may have prevented more significant results from being obtained, as some effects in a particular age range may have been diluted. Future studies, with a bigger sample size, should considered children of the same age (or a narrow age range). Additionally, the complexity of the video game task should be adapted to each age interval. This would allow us to observe whether the similar error variability we obtained in participants 13 years of age or older could be due to the fact that the presented driving task is simple for these children or whether, despite adapting the difficulty of the task to the age of the participants, performance variability is corroborated to decrease with increasing age.

Another limitation is that we have only focused on the study of attention. Future work could investigate how to use video games to study hyperactivity, impulsivity and other symptoms also in a continuous manner. It should be noted that some authors found that individuals with ADHD could be at increased risk for video game addiction [59]. We recommend moderating the use of gamification elements that increase engagement/addiction. A detailed explanation of types of video games and gamification rewarding elements can be found in [61].

Though participants’ rating was positive (7.81 out of 10), they complained about the length of the test. The task should be long enough to make it difficult for participants to maintain attention so that we can extract relevant information about when they begin to make more errors. As mentioned, future research could consider adapting the length of the tasks according to to the attention span of each child.

Our work provides a powerful method for studying attention. According to Bozhilova et al., individuals with ADHD compared to controls, showed greater mind wandering frequency with increasing inter-stimulus delays which suggests poor context regulation of mind wandering in response to increasing demands on sustained attention [62]. Future studies could be conducted with a larger sample to investigate the duration of attention/inattention periods and how this duration varies over time.

Some participants disliked the theme of the task. Themes could be adapted to children’s preferences. Additionally, having video games with different themes offers the possibility of performing apparently different tests to further observe the child’s behavior.

## 6. Conclusions

The main objective of this research is to study the possibilities offered by the analysis of the behavior of children diagnosed with ADHD throughout a whole test and the different performance patterns that appear for a more thorough knowledge of each child.

Our work demonstrates that it is possible to use a video game to obtain continuous measures of task performance. Aslo, collecting performance information data based on a video game allows for a gender-blind analysis. We propose using this information as a support to investigate attentional patterns over time in children already diagnosed with ADHD.

Although it would be necessary to repeat the study with a larger sample, the results suggest that there is a dependence between the age of the children and their ability to sustain attention.

The results of the trajectory and error plots show that different patterns of behavior occur and that it is possible to perform statistical analyses to classify different types of patterns.

We believe that video games can complement traditional tests by providing new information. The fact that our system was well rated (7.81 out of 10) encourages the use of similar approaches as an opportunity to gain deeper insight. Future work could explore how to use this information in conjunction with accurate diagnostic methods to achieve more personalized treatment, better suited to each child.

## Figures and Tables

**Table 1:** Results of the satisfaction questionnaire on the car driving system.

Rating	What Did You Like?	What Didn’t You Like?	Did You Find It Long?
Mean 7.81	Video game (38.60%)	Video game (8.77%)	Yes (59.65%)
SD 2.09	Car Theme (42.10%)	Car Theme (22.81%)	No (38.60%)
	No answer (19.30%)	Time (35.09%)	No answer (1.75%)
		Lack of realism (3.51%)	
		No answer (29.82%)	

## Data Availability

Not applicable.

## Appendix A. Sample Demographic Variables

**Table A1:** Sample Demographic Variables.

Age	Number	Female
7	1	0
8	2	2
9	4	3
10	7	2
11	8	1
12	7	1
13	10	1
14	6	3
15	1	0
16	7	2
17	10	4
